# Gluten-Free Labeling Is Misused Frequently in Foods Marketed in Northwestern Mexico

**DOI:** 10.3389/fnut.2021.687843

**Published:** 2021-06-25

**Authors:** Ana M. Calderón de la Barca, Valeria Luna-Alcocer, José R. Valenzuela-Miranda, Maria E. Mejía-León

**Affiliations:** ^1^Depto. Nutrición y Metabolismo, Centro de Investigación en Alimentación y Desarrollo, A.C. Hermosillo, Sonora, Mexico; ^2^Facultad de Medicina Mexicali, Universidad Autónoma de Baja California, Mexicali, Mexico

**Keywords:** gluten-free labeling, foods, celiac disease, analysis, mexican market

## Abstract

**Background:** Patients with celiac disease (CD) require a gluten-free (GF) diet, including industrialized products containing ≤ 20 mg gluten/kg. The market status of GF food products is almost unknown in Mexico. Therefore, we studied the GF-labeled products on the northwestern Mexican market and analyzed their gluten content.

**Methods:** We searched for GF type of foods in three different supermarkets of each chain in Mexicali Baja California and Hermosillo Sonora and corroborated the price, origin, and GF certification of each item using internet sites. We quantified the gluten in the foods using the sandwich R5-enzyme-linked immunosorbent assay (ELISA) and detected their immune-reactivity for IgA from patients with CD.

**Results:** The study included >263 different GF-labeled foodstuffs, and 55% of them were made in Mexico. The Mexican items were principally flours, sausages, bread and bakery, milk-type products, and tortillas, while pasta, snacks, and breakfast cereals were mainly imported. The cost ratio of GF products to the conventional mean was 3.5, ranging principally from 1 to 13. The most common GF-labeled foods were flours and pasta (34), cookies and snacks (32), breakfast cereals, sausages, and milk-type products (18–20). Although 36% of the products were certified, 17.4% of the analyzed samples contained >20 mg gluten/kg, mainly the non-certified ones and those made in Mexico. IgA from patients with CD reacted *in vitro* against gluten proteins from the contaminated GF-labeled products.

**Conclusion:** The accessibility of GF products in the northwestern Mexican market is wide; however, such products are expensive, and some could be risky for patients with CD because they contain gluten, which is recognized by the immune systems of these patients.

## Introduction

Wheat gluten proteins contribute viscoelasticity and extensibility to the dough used in the preparation of bread and other widely consumed foodstuffs all over the world. Although wheat-containing foods are innocuous for the majority of people, gluten exacerbates the signs and symptoms in those with celiac disease (CD), an autoimmune enteropathy that develops in ~1% of any population. Additionally, other wheat-related disorders (1), such as wheat allergies and non-celiac wheat sensitivity, affect 5–6% of the population; some of these disorders could be related to wheat proteins other than gluten. The food industry has developed dietary gluten-free (GF) foods for people suffering from CD and other intolerances. According to the *Codex Alimentarius*, GF foods consist of ingredients that do not contain wheat, rye, or barley, and the gluten level they contain must not exceed 20 mg/kg, in total, or <100 mg/kg in processed form (2).

In spite of the Code*x Alimentarius* and guidelines given by different governments regarding GF foodstuffs for their marketing, some of the products are not GF, as demonstrated for products in Spain (3), Italy (4), Turkey (5), India (6), and others. However, there is scarce information about the Mexican market for GF-labeled foods and their safety, and their regulation is deficient. The northwestern Mexican states of Baja California and Sonora both share borders with California and Arizona in the USA, and the residents used to cross the border to shop. Due to COVID-19, the border had been closed last year, and the northwestern markets were similar and could be representative of the markets throughout Mexico.

Therefore, the study aimed to describe and classify GF-labeled foodstuffs available in the northwestern Mexican market, compare their costs with those of their conventional counterparts, quantify the gluten in food products using the *Codex*-recommended enzyme-linked immunosorbent assay (ELISA) kit, and detect the immune-reactivity of IgA from patients with CD.

## Methods

### Collection of GF-Labeled Foods

First, all the GF-labeled food products found in the markets of the two capital cities of northwest Mexico, Hermosillo Sonora, and Mexicali Baja California, were registered from November 2020 to February 2021. Screening was carried out in three different supermarkets of each chain and specialized food shops in the two cities. Pictures were taken to verify the data for cost, quantity, origin, GF certification, and composition, from internet sites of the corresponding brands. Additionally, the costs of GF foods were compared with those of their conventional counterparts on a per weight basis. The total cost in terms of Mexican pesos and the grams of the presentation of each foodstuff were considered. With these data, the cost per 100 g product was calculated to make comparisons between GF and conventional products. Subsequently, it was calculated as a ratio of the relative cost of GF products with respect to their equivalent conventional ones, expressing the result as a GF/CONV ratio. We obtained the means and ranges of the cost ratio for different types of products.

The products were randomly selected and purchased for analysis of about 33% of each product type in order to reach a confidence level of 95%. The product types selected for this study include the following: breakfast cereals; oats and granola; pasta; cookies; flours; bread, bakery, and breading; sweet and salty snacks; and fresh and dried tortillas. The rest of the product types, such as sausages, dressings, or milk-type products, were not analyzed because they were not transported and produced in the same facilities as gluten-containing cereals or their derivatives.

After identification, a representative sample of each product was taken by quartering for analysis. Samples were finely ground in a kitchen blender, avoiding cross-contamination by careful washing and drying of the blender cup and accessories that come in contact with the sample after processing each sample. All samples were stored at −20°C until further analysis.

### Gluten Analysis

The GF foods were assayed by the Ridascreen Gliadin R7001 sandwich R5 ELISA, as proposed by the *Codex Alimentarius*. Each finely ground sample (250 mg) was extracted with 2.5 mL of a solution (cocktail) containing 250 mM 2-mercaptoethanol and 2 M guanidine hydrochloride in phosphate-buffered saline (7), following the recommendations of the manufacturer (R-Biopharm AG, Darmstadt, Germany). In products with chocolate or cocoa, 0.25 g of skimmed milk powder was added to the cocktail solution during the extraction procedure.

### SDS-Gel Electrophoresis, Electro-Blotting, and Immuno-Detection

Sample extraction was followed as described for ELISA analysis by Ridascreen Gliadin R7001. Electrophoresis and immuno-detection were carried out as previously described in the laboratory (8). Briefly, GF extracts were mixed (1:1, v/v) with ×5 extraction buffer (0.3 M Tris-HCl [pH 6.8], 0.35 M SDS, 50% [v/v] glycerol, 0.05% [w/v] bromophenol blue, 0.05% [w/v] β-mercaptoethanol, and 1,200 μL of water), vortexed for 20 min, heated at 95°C for 10 min, and centrifuged at 12,000 g for 10 min. The prepared samples were loaded onto 12% (w/v) polyacrylamide gels, and electrophoresis under denaturing and reducing conditions was performed at 200 V for 45 min. The gel was stained with Coomassie blue and silver stain, and the mirror gel was electro-transferred to membranes by semi-dry blotting. The membrane was incubated overnight with a 50x-diluted sera pool, from three patients with CD (two adults and one child), in TBST (50 mM Tris, 150 mM NaCl, 0.05% Tween 20, and 5 mM NaN_3_). After washing, incubation was conducted with HRP-conjugated rabbit anti-human IgA (DAKO, Glostrup, DK), 1:2,000 (v/v) in TBST, and then, the membrane was washed, and the HRP activity was developed with DAB (3,3′-diaminobenzidine tetrahydrochloride; Sigma, St Louis, MO) and the reaction was stopped by washing with water.

### Statistical Analysis

Descriptive statistics of the data were performed. Differences in cost between the GF-labeled products and their conventional counterparts were evaluated with paired *t*-tests. *P* < 0.05 were considered significant.

## Results

### Variety, Certification, and Costs

There were at least 263 different GF-labeled products in the northwestern Mexican market. The majority of them were the same in both studied locations (Sonora and Baja California) due to the common supermarket chains. As shown in Table 1, the most common GF products were flours and pasta, each one with 34 different products, followed by cookies and snacks, with 32 different products, and breakfast cereals, bread and bakery, sausages, and milk-type products, with around 18–20 different products each. Local or foreign institutions certified 36% of the total marketed GF-labeled foods. Two of the Mexican CD associations have certification lists accessible only for associates, with the following labels: ACELMEX and SSG (*Seguro Sin Gluten*); the rest are registered international certifications such as *Federación de Asociaciones de Celiacos de España* (FACE), European ELS, and the Gluten-Free Certification Organization (GFCO). The cost ratio of GF to conventional product mean was 3.5, ranging from 1 to 9, except for some flours and pasta products, which cost up to 20 times more than the corresponding conventional products.

**Table 1:** Gluten-Free (GF)-labeled product types, certification, and comparative costs with homologous conventional products (*n* = 263).

**Product type**	**Quantity**	**Certified *n* (%)**	**Cost ratio GF/CONV Mean (range)**	***p*-value**
Breakfast cereals	18	10 (55.5)	2.2 (1.0–5.0)	< 0.0001^*^
Oats and granola	14	8 (57.1)	3.0 (1.3 – 8.8)	0.0012^*^
Pasta products	34	23 (67.6)	7.5 (4.1–20)	< 0.0001^*^
Cookies	32	15 (46.8)	3.0 (1.0–9.3)	< 0.0001^*^
Grains	9	0	1.9 (1.0–2.8)	0.0005^*^
Flours	34	6 (17.6)	5.4 (1.0–2.8)	< 0.0001^*^
Bread, bakery and breading	18	0	4.3 (1.7–9.1)	0.0001^*^
Sweet and salty snacks	32	14 (43.7)	1.7 (1.1–3.4)	< 0.0001^*^
Fresh and dried tortilla	15	6 (40)	3.8 (1.0–13)	0.0263^*^
Sausages	20	2 (10)	1.4 (1.0–2.0)	0.0002^*^
Milk-type and milk foods	18	5 (27.7)	3.0 (1.0–6.1)	0.0014^*^
Sauces and dressings	12	3 (25)	2.7 (1.0–6.4)	0.0030^*^
Others	7	2 (28.5)	3.5 (1.2–9.0)	0.0357^*^

*GF, Gluten free; CONV, Conventional; n, sample size.*

* *Paired t-test (costs), p < 0.05 were considered significant*.

### Places of Origin and Brands

**Figure 1:**
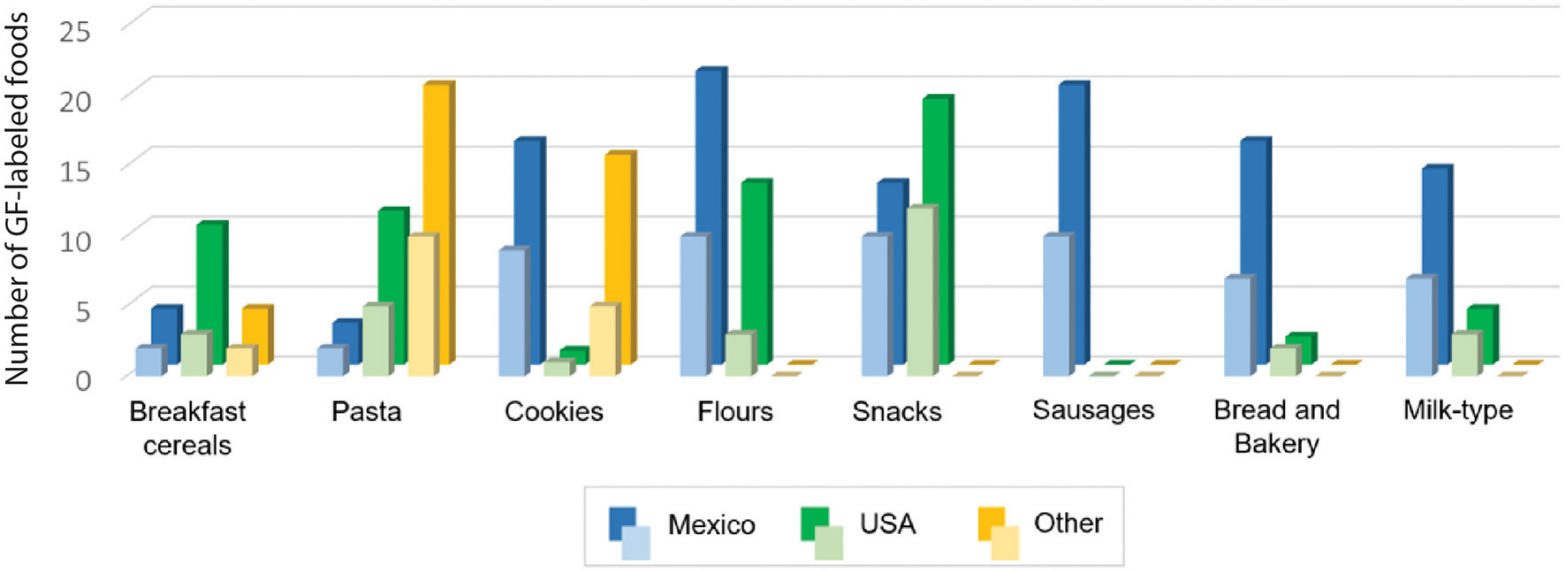
The total number of products (bold colors) and the number of different brands of each product type (light colors), according to their country of origin.

Figure 1 shows the places of origin of the most common GF-labeled products and their types in the studied markets, as well as the variety of brands per type and origin. While breakfast cereals, pasta, and snacks were principally imported products, cookies and flours were both Mexican and imported, and sausages, bread and bakery, and milk-type products were mostly Mexican. The imported products were mainly from the USA, followed by European countries, such as Spain, Italy, and Romania, and even some Latin-American countries, such as Ecuador and Costa Rica. Additionally, Figure 1 shows that most of the GF products are sold under a wide variety of brands, some of which only produce one or two different GF foodstuffs.

### Gluten Contamination

In respect to gluten contamination, Table 2 presents the nine analyzed product types, accounting for 206 different foodstuffs, from which a subsample of 86 products was analyzed for gluten content. Gluten was undetectable in 77% of the analyzed products but 5–16 mg/kg of gluten was detected in 6% of the products. Of the 86 food products analyzed, 15 (17.4%) had gluten contents >20 mg/kg, the majority of them originated in Mexico, one was from Ecuador, and another was from the USA (Table 2). Two of the contaminated samples presented with a GF certification by a Mexican CD association. Nine of the gluten-contaminated products contained between 20 and 100 mg/kg of gluten: one breakfast cereal product, two oat- and granola-type products, one pasta product, one presentation of cookies, three flour products, and one bread product. Three products (one cookie and two different brands of biscuits) contained 100–150 mg/kg gluten, while two bread and bakery products and one tortilla brand contained between 948 and 12,279 mg/kg of gluten. These last three products, as well as two of them with around 100 mg/kg gluten, were from a non-certified brand, which declared that potato and rice or corn flours with flaxseed ferment as their main ingredients. Another contaminated cake contained, according to its label, oat flour, banana, carrot, and several other minor grains. The gluten-contaminated breakfast cereal declared on its label quinoa and chia, with apple and cinnamon. The two oat products contaminated with gluten declared that they contained only oat flakes. The label of the gluten-containing pasta declared that it contained chickpea and chia. One of the gluten-contaminated cookies contained coconut, almond, cinnamon, and vanilla, and the other cookie product declared only rice, sugar, and additives. One of the gluten-contaminated flour products consisted of quinoa flour; there were two products with almond, oat, and rice flours, chocolate, and additives; and three products with oat, tapioca, and chickpea.

**Table 2:** Gluten contamination of GF-labeled products (*n* = 86).

**Product type**	**Analyzed products**	**Contaminated samples**	**Origin of contaminated samples**
Breakfast cereals	7	1	Ecuador
Oats and granola	6	2	Mexico
Pasta products	14	1	Mexico
Cookies	13	2	Mexico/USA
Grains	4	0	–
Flours	13	3	Mexico
Bread, bakery, and breading	9	5	Mexico
Sweet and salty snacks	13	0	-
Fresh and dried tortilla	7	1	Mexico

*n, sample size; USA, United States of America*.

### Reactivity of Human IgA Against Gluten in GF-Labeled Foods

**Figure 2:**
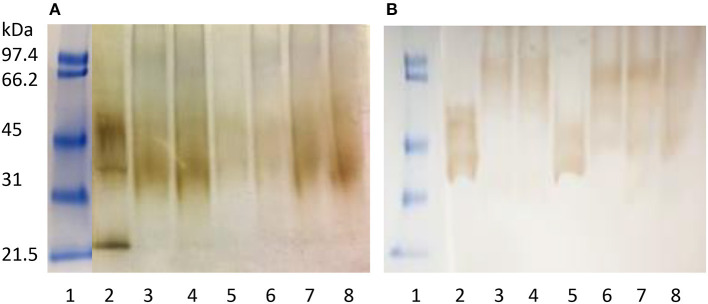
Immuno-reactive proteins in gluten-free (GF)-labeled foods detected with IgA of a pool of sera from three patients with celiac disease (CD). **(A)**: Coomassie blue and silver stained electrophoresis gel and **(B)**: blots after incubation with the sera pool. Lane 1: m.w.std., lanes 2–8: extracts of gluten contaminated foods with 40–12,279 mg/kg gluten.

Figure 2A shows the electrophoretic patterns of the proteins from seven GF-labeled foods with the highest gluten content stained with silver stain and Coomassie blue. All of them were extracted from the same quantity of product, and the sample in lane 2 appears to contain more protein with more defined subunits, given the intensity of the stain. Bands, although diffused (due to degradation by the food treatments) in lanes 3–8, could correspond to subunits of gliadins (35–50 kDa and 66 kDa). IgA in the sera from patients with CD clearly and differentially reacted to the gluten proteins from GF-labeled foods electro-transferred to the membrane and present in lanes 2–8 (Figure 2B), in agreement with the ELISA-based gluten assay. Although all the samples in part A show mainly protein subunits between 35 and 50 kDa, the blot in part B shows a differential pattern. While IgA from patients with CD recognized the same 35–50 kDa subunits of prolamins in samples from lanes 2 and 5, in samples 3, 4, 6, 7 and 8, they recognized higher molecular weight protein subunits. Samples from lanes 2 and 5 contained oats, while the rest of the samples were from the same brand and it did not declare any more that potato and rice or corn flours for all the its products.

## Discussion

The total number of GF products in the Spanish market exceeds that included in this research by a factor of 8.5 (2,247 vs. 263) (3). A possible reason is that CD emerged in Mexico a short time ago; only 8 years ago, it was still considered as rare (9). Although some GF-labeled breakfast cereals, oats, grains, and cookies were comparable in cost with their conventional counterparts, in general, GF foods were more expensive. Bread and bakery foodstuffs, pasta products, and flours were on average 4.3–7.5 times more expensive than the equivalent conventional products, although some products were up to 20 times more expensive. In general, imported GF foods were the most expensive, but some Mexican products, such as pasta, were also expensive. The mean cost ratios of GF to conventional foods in this study (1.4–7.5) were considerably higher than those reported in Greece (1.2–3.4) (10) and similar to those reported in Spain (1.3–6.9) (3).

Certified and imported GF products contained < 20 mg/kg of gluten except two foreign products and two certified Mexican foodstuffs (Table 2). According to the published list of ACELMEX-certified products, some of the brands were formerly certified for GF-labeling, but, currently, they have not renewed their certification. There were common ingredients, such as oats, rice, and quinoa, in some of the gluten-contaminated products. Perhaps the foodstuffs were prepared with some cross-contaminated cereal flours of 20–100 mg/kg, such as those containing oats and rice. The quantity of 948–12,279 mg/kg of gluten corresponds to 1–10% of wheat flour in the total mix, and it is not cross-contamination. Electrophoretic patterns and the recognition of gluten proteins by IgA from patients with CD, as shown in Figure 2A, demonstrated that there were gluten proteins in some of the analyzed GF-labeled foods. Although the subunits of higher m.w. (around 66 kDa) were represented by faded spots in Part A, the IgA from patients with CD clearly recognized them in Part B because they are the most immunogenic wheat proteins (8). The bread, cake, and tortilla with the highest gluten content and immune reactivity of IgA in patients with CD were from a popular brand with several bakeries and cafeterias in different Mexican cities, in addition to not having any GF certification.

Interestingly, none of the snacks were contaminated with gluten, and the salty ones were mainly fried products. According to Thompson et al. (11), ELISA may underperform when used on heat-treated samples; however, the sandwich-type ELISA combined with extraction using the cocktail solution employed in this study performed well enough to detect gluten contamination in fries analyzed by this method. Additionally, the cocktail solution developed by García et al. (7) has demonstrated to be an excellent extraction procedure.

The percentage of GF-labeled foods in northwest Mexico that were gluten contaminated amounted to 17.4%, with a mean of 1,580 (range: 30–12,279 mg/kg) mg/kg, considerably higher than percentages and means obtained for GF-labeled foods in other locations. In Italy, GF-labeled products with >20 mg/kg of gluten were uncommon (9%) and quantitatively low with a mean of 59 mg/kg (4). In southern India, 9.8% of the products contained > 20 mg/kg of gluten with a mean of 32.5 mg/kg (6). In Turkey, 17.5% of the analyzed GF-labeled samples contained > 20 mg/kg of gluten, although it was principally due to the use of buckwheat flour (5). It is clear from this study that, if we discard the brand with the most contaminated products, the results are comparable with those previously discussed. We hope that GF-labeled foods will improve after strict Mexican regulations are put in place.

As in other countries and given these results, the main concern is the marketing of food products that are GF-labeled, without the necessary tests and certification. This is especially important when the products have natural GF grains as ingredients and are assumed to be safe (12). The only commercially available test approved by the *Codex Alimentarius* is the ELISA R5 test, which was used in this study. The nutrition label information is the only guide that people with CD and other wheat-related diseases have when choosing different foods. The labeling should serve for consumer protection and not constitute a risk, as happened with some of the products evaluated in this study, when the labeling contained unverified information and did not have an official certification that supports the contents containing below the 20 mg/kg of gluten. As a recommendation, it is considered essential that the health professional contributes to the education of patients to learn how to identify, verify, and choose only those GF products that have an official certification, and not based on the possibly misleading labeling information.

The Mexican Health Secretariat is currently updating the official standard NOM-086 on food labeling in compliance with the *Codex Alimentarius*, in order to protect the general population and patients with CD and other patients with wheat-related diseases and to provide safe GF-labeled manufactured food products. This standard underscores the role of the government in the enforcement of the GF certification of these products. The implementation of the regulation and its effective application would allow better control of patients with CD, as well as diversify the options of products supported by certification. This could help reduce costs in the medium and long term, which is another drawback of these types of products (13). In addition to Mexico, other Latin American countries, such as Argentina, Chile, and Brazil, have begun to modify the laws to regulate the labeling of GF products in the last decade (13). However, some of these regulations do not define tolerance limits for gluten content or mention control measures. In Mexico, it is necessary to verify the effective adherence of the food product manufacturing industry to guarantee safety.

In conclusion, the accessibility of GF-labeled industrialized products in the northwest Mexican market is sufficient, although the majority of such products are expensive, with 45% of them being imported from several countries, mainly the USA. However, some of them could be risky for patients with CD due to their gluten content. Brand regulation over the use of GF-labeling is urgently required.

## Data Availability Statement

The raw data supporting the conclusions of this article will be made available by the authors, without undue reservation.

## Author Contributions

AMC and MM-L designed and conducted the research. AMC wrote the first manuscript version. VL-A made the market study and selected the analyzed sample. VL-A and JV-M carried out all the analyses. All authors read the manuscript and agreed to be accountable for the content of the work.

## Conflict of Interest

The authors declare that the research was conducted in the absence of any commercial or financial relationships that could be construed as a potential conflict of interest.
